# Anti-PEG Immunogenicity of mRNA-LNP Vaccines in Humans: Evidence for Population-Level Changes in the Anti-PEG Antibody Repertoire

**DOI:** 10.3390/pharmaceutics18070815

**Published:** 2026-06-30

**Authors:** Réka Facskó, Tamás Mészáros, Petra Berényi, Zsófia Szabó, János Szebeni, Gergely Tibor Kozma

**Affiliations:** 1 Nanomedicine Research and Education Center, Institute of Clinical Pathophysiology, Semmelweis University, 1085 Budapest, Hungary; facskoreka@gmail.com (R.F.); meszaros.tamas@semmelweis.hu (T.M.); berenyipetra@gmail.com (P.B.); jszebeni2@gmail.com (J.S.); 2 SeroScience LLC, 1085 Budapest, Hungary; 3 Department of Laboratory Medicine, Semmelweis University, 1085 Budapest, Hungary; szabo.zsofia2@semmelweis.hu

**Keywords:** anti-PEG antibodies, polyethylene glycol (PEG), polysorbate (PS), lipid nanoparticles (LNPs), mRNA vaccines, antibody avidity, affinity maturation, hypersensitivity reactions (HSRs)

## Abstract

**Background/Objectives**: Polyethylene glycol (PEG) is widely used to enhance the stability and pharmacokinetics of nanomedicines, including lipid nanoparticle (LNP)-based mRNA vaccines. However, both pre-existing and vaccine-induced anti-PEG antibodies may compromise the efficacy and safety of PEGylated therapeutics. **Methods**: In this study, we analyzed the specificity and avidity of anti-PEG antibodies in human blood donor samples from Unvaccinated individuals and recipients of PEGylated mRNA-LNP vaccines (Comirnaty and Spikevax), polysorbate-containing vaccines, or vaccines lacking both PEG and polysorbates. Quantitative ELISA was used to characterize anti-PEG and anti-polysorbate IgM and IgG responses in 325 plasma samples, while an equilibrium titration method was applied to assess IgG binding to PEG molecules, micelles, and PEGylated liposomes with defined structural features in 36 plasma samples. **Results**: Vaccination with PEGylated mRNA-LNPs was associated with increased anti-PEG antibody levels and qualitative changes in antibody binding behavior. Anti-PEG IgG antibodies displayed progressively higher avidity toward larger and structurally more complex PEG-containing antigens, with the strongest binding observed for PEGylated liposomes. Notably, vaccinated individuals, particularly those who received Spikevax, showed increased end-group and backbone-specific avidity, as well as an enhanced ability of antibody paratopes to engage shorter PEG chains. In contrast, polysorbate-containing or PEG-free vaccines did not elicit comparable effects. **Conclusions**: These findings suggest that vaccination with PEGylated mRNA-LNPs is associated with the emergence of altered antibody populations with increased end-group reactivity and higher avidity toward PEG-directed immune responses, despite PEG being a synthetic, nonprotein polymer. Antibody binding to LNPs, accompanied by the emergence of high-avidity anti-PEG IgG seems to be consistent with an increased risk of adverse events, particularly following repeated vaccinations, including complement activation-related pseudoallergy (CARPA) and anaphylaxis. It may also contribute to altered immune protection against the vaccine target, underscoring the need for avidity-aware risk-benefit assessment of PEGylated therapeutics.

## 1. Introduction

Polyethylene glycol (PEG) is a synthetic polyether formed by polymerization of ethylene oxide, yielding molecules of variable chain lengths and molecular weights that can possess different terminal end-groups. Owing to its biological inertness, low toxicity, and biocompatibility, PEG has been classified by the U.S. Food and Drug Administration (FDA) as Generally Recognized as Safe (GRAS) [1]. Polysorbates (PSs) (e.g., PS80 and PS20) are commonly used pharmaceutical surfactants that share structural similarity with PEG, featuring poly(oxyethylene) chains attached to a sorbitan ester head group [2]. The PEG sheath reduces opsonization by plasma proteins and macrophage uptake, thereby prolonging circulation time and enhancing pharmacokinetics and therapeutic efficiency [3,4,5,6]. PS80 is widely used as an emulsifier and stabilizer in vaccines [7]. Both PEGs and PSs are also present in cosmetics and food products, resulting in continuous, low-level human exposure.

In 2020, the first PEGylated lipid nanoparticle (LNP)-based mRNA formulations received FDA approval as COVID-19 vaccines: Comirnaty (Pfizer Inc. (New York, NY, USA)/ BioNTech Manufacturing GmbH (Mainz, Germany), BNT162b2) and Spikevax (Moderna Inc. (Cambridge, MA, USA), mRNA-1273). Both vaccines contain methoxy-terminated PEG2000 chains (mPEG2000), although these are conjugated to different lipid anchors: dimyristyl groups linked to PEG via an acetamide spacer (ALC-0159) in Comirnaty, and dimyristoyl glycerol (DMG) in Spikevax [8]. While PEGylated therapeutics had been used before, these vaccines represent the first global-scale administration of PEGylated formulations, affecting billions of people, resulting in unprecedented population-wide exposure to PEG. In contrast, adenoviral vector-based COVID-19 vaccines, such as AstraZeneca, Sputnik V, and Janssen, contain PS80 as a stabilizing excipient, which is not covalently attached to the viral vector [7].

Even though PEG was long considered biologically inert, antibodies against it have been detected with a reported prevalence ranging from approximately 50% to 97.5% for at least one antibody isotype, even among healthy individuals without prior exposure to PEG-containing therapeutics [9,10,11,12,13]. This wide variability, in addition to natural variations among subjects, is attributed to differences in assay sensitivity and specificity. In our previous study, we reported a high prevalence of anti-PEG antibodies in healthy donors, with detectable anti-PEG IgG in 98% and anti-PEG IgM in 99% of individuals [14]. Due to the structural similarities between PEGs and PSs, specific antibodies against them are often highly cross-reactive [15,16,17]. These pre-existing or naturally occurring IgM and IgG antibodies against PEG and PS may reduce the bioavailability and efficacy of PEG-containing therapeutics. Additionally, they can affect the safety of PEGylated therapeutics by activating the complement system, leading to the release of anaphylactic factors, causing hypersensitivity reactions (HSRs) referred to as complement activation-related pseudoallergy (CARPA) [18,19,20,21].

Following vaccination with Spikevax or Comirnaty, several studies [14,22,23,24,25] reported significant increases in anti-PEG IgM and IgG levels, potentially modifying PEG-related immune effects. Importantly, these effects may depend not only on antibody concentration but also on antibody avidity (describing the affinity of the multiple interactions) and specificity toward PEGs and PSs [26]. While anti-PEG antibody concentrations are commonly used to assess immunogenicity, high-avidity antibodies may form more stable interactions with PEGylated surfaces and may thereby enhance opsonization, immune complex formation, and complement activation rather than low-avidity antibodies present at similar concentrations [26,27,28].

Antibody recognition is likely influenced by the structural design of PEGylation, including PEG chain length, oxyethylene repeat number, molecular conformation, surface density, and architecture, which are critical determinants of the stealth and stabilization properties of PEGylated therapeutics [29]. However, the contribution of these structural features to anti-PEG antibody binding remains poorly understood. Our hypothesis was that in PEGylated mRNA-LNP vaccinated samples, anti-PEG antibody levels not only increased but also underwent qualitative changes, including altered avidity, end-group recognition, and structural binding preferences.

The avidity of anti-PEG IgG antibodies toward PEG-OH molecules, PEG micelles, and PEGylated liposomes was analyzed in samples from Unvaccinated and COVID-19 mRNA-LNP-vaccinated donors [30,31]. In addition, a larger cohort was used to quantify IgM and IgG antibody levels against PEG and PS and to evaluate antibody binding preferences across different COVID-19 vaccination backgrounds.

The study provides a comprehensive analysis of anti-PEG antibodies’ avidity and specificity for structurally diverse PEG antigens and COVID-19 mRNA-LNP vaccination. Our findings highlight that the vaccination seems to reshape the anti-PEG antibody repertoire toward higher avidity and to enhance recognition of methoxy-terminated PEG structures. Since anti-PEG antibodies have been implicated in hypersensitivity reactions to PEGylated mRNA-LNP vaccines [32,33,34,35,36,37,38], the immunological response to PEG-containing therapeutics in the current population may no longer reflect patterns observed before the worldwide use of COVID-19 mRNA-LNP vaccines. Understanding these qualitative changes is therefore essential for predicting the safety and performance of future PEGylated therapies.

## 2. Materials and Methods

### 2.1. Materials

Sodium bicarbonate, sodium carbonate, 3-[(3-Cholamidopropyl) dimethylammonio]-1-propanesulfonate hydrate (CHAPS), bovine serum albumin (BSA), polysorbate20, 3,3′,5,5′-tetramethylbenzidine (TMB), phosphate-buffered saline (PBS), polyethylene glycol molecules with a hydroxy end-group, sulfuric acid, and peroxidase-conjugated goat polyclonal anti-human IgM and IgG were from Sigma Chemical Co. (St. Louis, MO, USA). The Polysorp Nunc-Immuno Plate was from Thermo Fisher Scientific (Waltham, MA, USA). Humanized recombinant anti-PEG IgM and IgG were from Academia Sinica (Taipei, Taiwan). Cholesterol and 1,2-distearoyl-sn-glycero-3-phosphoethanolamine-N-[methoxy(polyethylene glycol)-2000] ammonium salt (DSPE-mPEG2000) were purchased from Avanti Polar Lipids Inc. (Alabaster, AL, USA). Hydrogenated soy phosphatidylcholine (HSPC) was from Lipoid Inc. (Ludwigshafen, Germany). All lipids had a purity of ≥97%.

### 2.2. Blood Samples

For the ELISA equilibrium titration measurement to define the anti-PEG antibodies’ avidity, 36 plasma samples (11 Unvaccinated, 17 Comirnaty-, and 8 Spikevax-vaccinated) were selected from the larger study cohort. Sample selection was purposive, as the assay required relatively large plasma volumes and sufficiently high anti-PEG IgG concentrations (>20,000 ng_eq_/mL) to enable reliable competitive ELISA measurements. The characteristics of the selected samples are summarized in Supplementary Table S1.

To analyze the IgM and IgG antibodies against PEG and PS antigens, 325 blood plasma samples were studied: 114 Unvaccinated, 146 PEGylated LNP-vaccinated (Comirnaty, Spikevax), 37 PS80 containing COVID-19-vaccinated (AstraZeneca and Sputnik V), and 28 neither PEG- nor PS-containing COVID-19-vaccinated (Sinopharm). Groups for the statistical analysis, types, and numbers of vaccines received by donors are shown in Table 1.

All participants or parents of minors filled out a consent form and questionnaire about their demographic information, previous SARS-CoV-2 infection, COVID-19 vaccination history, adverse reactions following vaccination, use of consumer products containing PEG or polysorbates, previous medical interventions involving PEG- or polysorbate-containing products, and allergic diseases. Plasmas were separated by centrifugation and stored at −80 °C until analysis. The study was approved by the Scientific and Research Ethics Committee of the Hungarian Medical Research Council (52685-6/2022/EÜIG).

### 2.3. Enzyme-Linked Immunosorbent Assays (ELISAs) for Anti-PEG and Anti-PS Antibodies

Four different ELISAs were used to quantify plasma IgM and IgG levels against PEG and PS. Binding specificity was quantified by the ratio of PS-specific to PEG-specific antibody concentrations. ELISAs were performed based on a previously described method [19,39], where the PEG or PS specificity of measured antibodies was guaranteed mainly by DSPE-mPEG2000 or PS20 coating, respectively.

In short, Polysorp (Nunc) plates were coated overnight at 4 °C with DSPE-mPEG2000 to measure anti-PEG IgM and IgG, and with PS20 to measure anti-PS IgM and IgG. Wells were washed three times for 1 min with wash buffer containing PBS/0.05% CHAPS in anti-PS IgM and IgG and anti-PEG IgG measurements and with PBS/0.05% PS20 in anti-PEG IgM measurement, followed by blocking of the wells with wash buffer supplemented with 2% bovine serum albumin (BSA) at 37 °C for 1 h. Different wash buffer compositions were used for the assays to minimize nonspecific background signals and optimize assay performance. Plasma samples, as well as anti-PEG IgG and IgM reference standards, were diluted in the wash buffer supplemented with 1% BSA (dilution buffer) and incubated in the wells for 1.5 h at 37 °C, with slow shaking. Wells were washed five times with wash buffer as described earlier. After staining with HRP-conjugated anti-human IgM or IgG (depending on the measurement target) for 1 h, wells were washed again five times with wash buffer as mentioned. Wells were stained by incubation for 15 min with substrate solution (Neogen) containing 3,3′,5,5′-tetramethyl benzidine (TMB) and hydrogen peroxide. The reaction was stopped with 2 N H_2_SO_4_, and A450 was read with a Fluostar Omega 96-well plate reader (BMG Labtech, Ortenberg, Germany). Antibody concentrations were determined by interpolation from standard curves generated using serial dilutions of dedicated human plasma samples stored in aliquots at −80 °C. The same aliquoted reference plasma standards were used throughout the study and stored under identical conditions. The curve-fitting model was selected based on assay validation and was applied consistently across assay runs. First-order curve fitting was used for assays performed with PS20-containing washing and blocking buffers, whereas second-order curve fitting was used for assays performed with CHAPS-containing wash buffers.

### 2.4. Antigen Preparation for Competition Assays

Hydroxy PEG (PEG-OH) molecules, PS20 or DSPE-mPEG2000 micelles, and liposomes (conventional or PEGylated) were used as competitor antigens in the ELISA equilibrium titration method (see below). Molecules and micelles were prepared simply by dissolving the corresponding components in PBS. Liposomes were prepared using the same method as it was described earlier for the preparation of 5 mol% PEG-containing liposomes [40]. In brief, freeze-dried lipid components (fully hydrogenated soy phosphatidylcholine (HSPC), cholesterol (Chol), DSPE-mPEG2000) were hydrated in 10 mL sterile pyrogen-free normal saline based on molar ratios (Table 2) by vortexing for 2–3 min at 70 °C to form multilamellar vesicles (MLVs). The MLVs were downsized through 0.4 and 0.1 μm polycarbonate filters in two steps, 10 passes through each, using a 10 mL extruder barrel from Northern Lipids (Vancouver, BC, Canada) at 62 °C. Liposomes were suspended in 0.15 M NaCl/10 mM histidine buffer (pH 6.5), and their sizes were measured using Zetasizer Nano S (Malvern).

### 2.5. ELISA Equilibrium Titration

To calculate the inhibition curves and the average equilibrium constant (*K*) of anti-PEG IgG antibodies against different PEG-containing antigens, our anti-PEG IgG ELISA method (described above) was extended with the ELISA equilibrium titration method described by Friguet et al. [30]. Briefly, each human plasma sample was diluted with the ELISA’s dilution buffer to contain the same amount (2000 ng_eq_/mL) of anti-PEG IgG (the highest concentration that can be measured by our direct ELISA), and aliquots were mixed with an equal volume of dilution buffer containing PEG competitor antigens in various concentrations. The competitor concentration range was titrated before assays for each type of competitor antigens (see in detail Table 3). Mixtures were incubated for 1 h at 37 °C with slow shaking in polypropylene plates, meanwhile the pre-coated and washed Polysorp plates were also incubated with wash buffer supplemented with 2% bovine serum albumin (BSA) for blocking under the same conditions. After the parallel incubations, the mixtures from polypropylene plates were transferred to the blocked Polysorp ELISA plates, and our anti-PEG IgG assay was continued as described above. During the measurement, an equilibrium occurred between anti-PEG antibodies bound to soluble PEG competitor antigens and PEG immobilized on the ELISA plate, and the plate-bound fraction was subsequently quantified.

### 2.6. Calculate the Average Equilibrium Constant (K) and the Number of PEG Molecules Required to Achieve 50% Inhibition of Anti-PEG IgG Binding

To calculate the average equilibrium constant (*K*) from the ELISA equilibrium titration results, we used a linearization method by Bobrovnik et al. [31], which describes the linear dependence between the value of the algebraic expression on the left-hand side, and the concentration of the antigen $l_{i}$:

(1) $$\frac{c_{0}-c_{i}}{c_{i}}+\;\sqrt{{\left(\frac{c_{0}-c_{i}}{c_{i}}\right)}^{2}+\frac{c_{0}-c_{i}}{c_{i}}\;}=K*l_{i}$$

where $c_{0}$ is the anti-PEG IgG antibody concentration in the absence of competitor antigen, and $c_{i}$ is the anti-PEG IgG antibody concentration in the presence of competitor antigen at $l_{i}$ concentration. *K*, the slope of the linear dependence, shows the value of the average equilibrium constant, representing the average avidity of PEG-specific polyclonal antibodies against the given PEG-containing antigen.

We quantified only anti-PEG IgG antibodies; however, the analyzed plasma samples contained antibodies of multiple isotypes. Anti-PEG IgE has been reported in rare cases and is predominantly cell-bound, making its contribution to equilibrium titration-based avidity measurements negligible [32,41,42]. In our previous studies, anti-PEG IgG levels were substantially higher than anti-PEG IgM concentrations in both vaccinated and Unvaccinated individuals [14]. Although IgM antibodies can exhibit high apparent avidity due to their pentameric structure, steric constraints may limit efficient multivalent engagement under equilibrium conditions. Therefore, the observed binding behavior predominantly reflects anti-PEG IgG interactions.

The number of PEG molecules required to reduce antibody binding by 50% was determined from inhibition curves. In ELISA equilibrium titration experiments, anti-PEG IgG concentrations were measured in the presence of increasing molar concentrations of hydroxy-terminated PEG competitors (PEG1500, PEG2000, and PEG4000). Percentage inhibition was calculated by comparing the measured anti-PEG IgG concentration in the presence of competitor to that obtained in the absence of competitor. Inhibition curves were generated by plotting percentage inhibition against the number of PEG molecules, which was calculated from the molar concentration of the antigen.

The average equilibrium constant (*K*) and the concentration of competitor antigen required to achieve 50% inhibition describe related but different aspects of antigen–antibody interactions. The latter is derived from a single point on the inhibition curve and reflects the apparent competition efficiency. In contrast, the equilibrium constant is calculated by fitting the entire binding curve using a model that accounts for IgG bivalency and multivalent antigen presentation, thereby integrating binding behavior across a broad concentration range [31,43].

### 2.7. Statistical Analysis

The statistical analysis was performed by GraphPad Prism 9 software. All log-transformed data showed a normal distribution according to the D’Agostino–Pearson test. Statistical significance was determined using one-way or two-way analysis of variance (ANOVA), as appropriate, followed by Tukey’s, Dunnett’s, or Šídák’s multiple comparisons tests. All analyses were performed on log-transformed data. *p* < 0.05 was considered statistically significant (* *p* < 0.05; ** *p* < 0.01; *** *p* < 0.001; **** *p* < 0.0001).

## 3. Results

### 3.1. Assessing PEG Specificity in Anti-PEG Antibody Measurements

Since PEGylated liposomes are composed of multiple components (Table 2), it was necessary to ensure that the measured changes in anti-PEG antibody concentration in competition experiments with PEGylated liposomes were specifically caused by PEG molecules. To verify this, control experiments were performed using conventional (non-PEGylated) liposomes in the ELISA equilibrium titration method [30]. These liposomes are similar to PEGylated liposomes in size distribution and composition, with the exception of the DSPE-mPEG2000 component (Table 2). Conventional liposomes did not affect the measured anti-PEG antibody concentrations (Supplementary Figure S1), confirming that the decrease observed in subsequent experiments with PEGylated liposomes was specifically attributed to PEG molecules present on the surface of the liposomes.

### 3.2. Avidity of Anti-PEG Antibodies in Unvaccinated Donors

All tested PEG-containing antigens (within the concentration ranges shown in Table 3) successfully competed with plate-bound DSPE-mPEG2000 for PEG-specific antibodies in donors’ plasma samples. Linear relationships were obtained when the left-hand side of Equation (1) was plotted against competitor antigen concentration ($l_{i}$), indicating that, despite the expected heterogeneity of polyclonal anti-PEG antibodies, the calculated equilibrium constant represents an average value reflecting the combined contribution and relative proportions of antibodies with different affinities [30,31].

**Figure 1:**
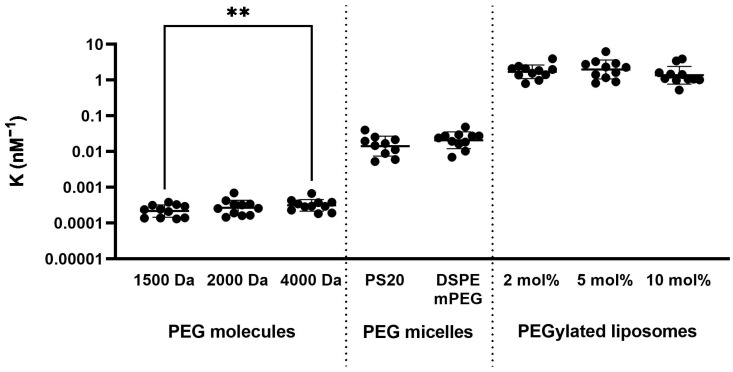
Average equilibrium constant (*K*) of pre-existing anti-PEG IgG antibodies for different competitor antigens. Measurements were performed using plasma samples from Unvaccinated donors and included three PEG molecules of different lengths, two types of PEG micelles, and three PEGylated liposomes containing different amounts of PEG chains. Data points represent log-transformed *K* values of donor samples on a logarithmic *y*-axis with back-transformed values shown on the scale. Statistical significance was determined by one-way repeated measures ANOVA followed by Tukey’s multiple comparisons test on the log-transformed data. *p* < 0.05 was considered statistically significant (** *p* < 0.01). Differences in *K* between the different types of competitor antigens (molecule, micelle, liposome) were significant in all cases (*p* < 0.0001).

As shown in Figure 1, the anti-PEG antibody avidity increased with the hydrodynamic size of the competitor antigen. PEG-OH molecules exhibited the lowest avidity with the smallest hydrodynamic size, as they are present as free molecules in aqueous solution. These molecules contain hydroxyl end-groups, which lack distinct terminal moieties; therefore, binding primarily reflects recognition of the PEG backbone. Among commonly used PEG end-groups, hydroxyl-terminated PEG exhibits the lowest binding affinity [44,45]. The average equilibrium constant for 4000 Da PEG-OH was significantly higher (*p* = 0.0013) than for 1500 Da PEG-OH, indicating stronger antibody binding to longer PEG chains.

Anti-PEG antibodies exhibited significantly higher avidity (*p* < 0.0001) for PS20 and DSPE-mPEG2000 micelles than for PEG-OH molecules. Despite differences in size (DSPE-mPEG2000 micelles Z(average) 16.8 ± 0.3 nm [46]; PS20 micelles Z(average) 7.2 nm [47]) and end-group composition between PS20 and DSPE-mPEG2000 micelles, no significant difference in anti-PEG antibody avidity was observed.

Among all tested PEG-containing competitor antigens, PEGylated liposomes exhibited the highest anti-PEG IgG avidity (*p* < 0.0001). These structures had the largest hydrodynamic size (Table 2) and were estimated to contain thousands of surface-anchored PEG chains (Table 3). Although no significant difference in avidity was observed among liposomes with different PEG contents, a trend toward increased avidity was seen when PEG density increased from 2 to 5 mol%, followed by a decrease from 5 to 10 mol%, indicating a non-monotonic relationship between PEG density and antibody avidity.

**Figure 2:**
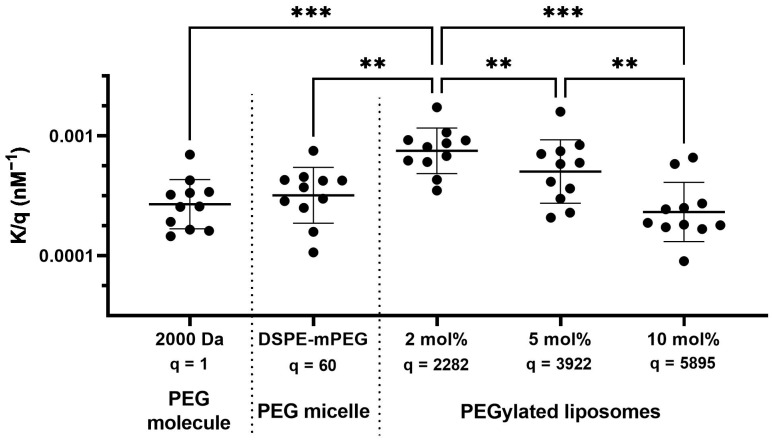
Apparent relative average equilibrium constant (*K*/*q*) of anti-PEG antibodies to 2000 Da PEG molecules presented on different types of competitor antigens. Average equilibrium constants (*K*) were normalized by the number of estimated PEG-related molecules per structure (*q*) for 2000 Da length PEG-containing competitor antigens. Data points represent log-transformed *K*/*q* values of Unvaccinated donor samples on a logarithmic *y*-axis with back-transformed values shown on the scale. Statistical significance was determined by one-way repeated measures ANOVA followed by Tukey’s multiple comparisons test on the log-transformed data. *p* < 0.05 was considered statistically significant (** *p* < 0.01; *** *p* < 0.001).

To further assess PEG-dependent binding, the equilibrium constant per individual PEG molecule (*K/q*; *q* is presented in Table 3) was calculated for all competitor antigens containing 2000 Da PEG chains in Unvaccinated donors’ plasma (Figure 2). Using this approach, no significant difference was observed in anti-PEG antibody avidity between free PEG-OH molecules and PEG chains presented in DSPE-mPEG2000 micelles. In contrast, PEG chains presented on liposomes containing 2 mol% PEG exhibited the highest relative avidity, whereas increasing PEG density resulted in a progressive decrease in avidity per PEG chain, reaching the lowest value at 10 mol%. Since avidity was normalized to total PEG content, reduced accessibility of PEG chains at higher PEG densities may partially contribute to this trend.

### 3.3. Effect of mRNA-LNP Vaccinations on Anti-PEG Antibody Avidity

**Figure 3:**
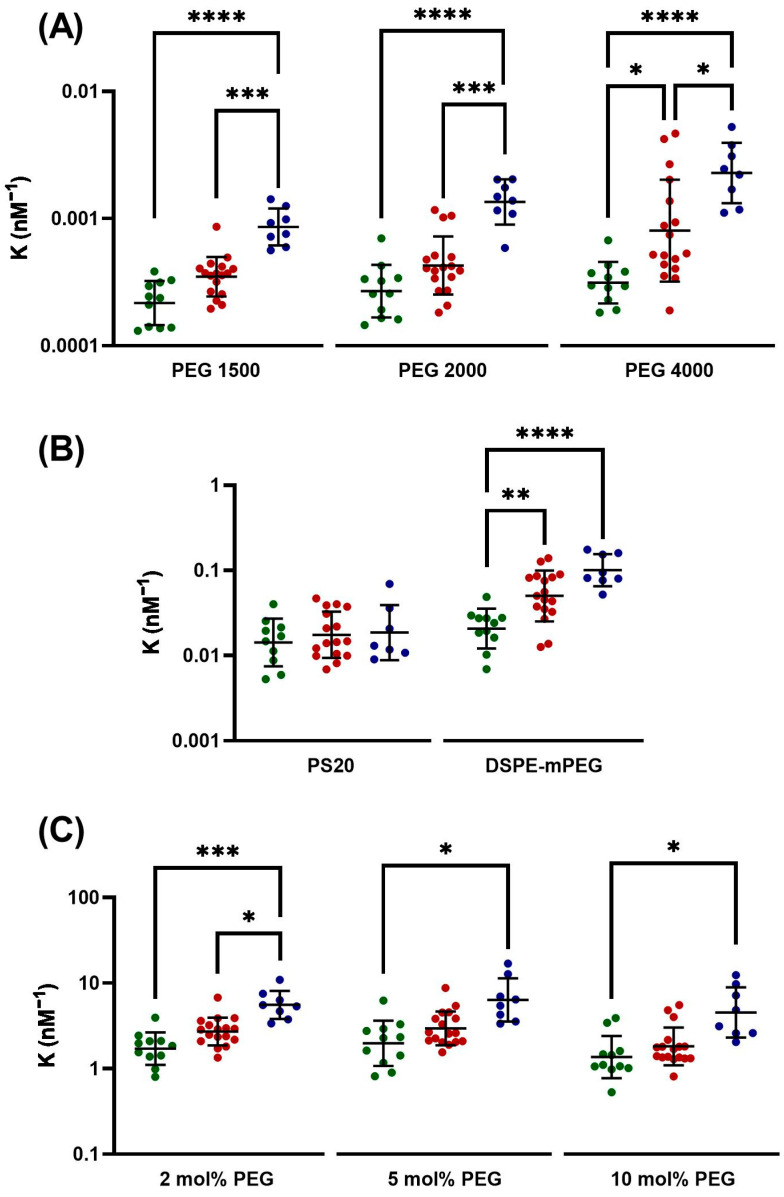
Average equilibrium constant (*K*) of anti-PEG antibodies in PEGylated LNP-vaccinated and Unvaccinated donors. Data points represent *K* values from Unvaccinated (green), Comirnaty-vaccinated (red), and Spikevax-vaccinated (blue) donors, measured using three different lengths of PEG molecules (**A**), two different types of PEG micelles (**B**), and three PEGylated liposomes containing different amounts of PEG (**C**). Log-transformed *K* values are on a logarithmic *y*-axis with back-transformed values shown on the scale. Statistical analysis was performed on log-transformed *K* values using two-way repeated-measures ANOVA followed by Tukey’s multiple-comparisons test. *p* < 0.05 was considered statistically significant (* *p* < 0.05; ** *p* < 0.01; *** *p* < 0.001; **** *p* < 0.0001). For multiple-comparison correction, all competitor antigens within each antigen class were analyzed as a single family. Accordingly, nine comparisons were performed for PEG-OH molecules (**A**), nine for PEGylated liposomes (**C**), and six comparisons were performed for PEG micelles (**B**).

Figure 3 compares the avidity of anti-PEG antibodies in plasma samples from Unvaccinated, Comirnaty- and Spikevax-vaccinated donors, using different PEG-containing competitor antigens. The avidity of anti-PEG antibodies toward all three tested PEG-OH molecules was significantly higher (*p* < 0.0001) in samples from Spikevax-vaccinated donors compared with Unvaccinated controls (Figure 3A). A similar trend was observed in Comirnaty-vaccinated donors; however, statistically significant differences were detected only for the 4000 Da PEG-OH antigens. These findings indicate that both mRNA-LNP vaccines induce anti-PEG IgG antibodies with increased avidity toward PEG-OH molecules compared with the pre-existing antibodies in Unvaccinated individuals. Moreover, antibody avidity toward all three tested PEG-OH molecules was significantly higher in Spikevax-vaccinated than in Comirnaty-vaccinated donor samples.

No significant differences were observed in anti-PEG IgG avidity toward PS20 micelles between the Unvaccinated and vaccinated groups (Figure 3B). In contrast, significantly higher avidity was detected for DSPE-mPEG2000 micelles in plasma samples from both Comirnaty- and Spikevax-vaccinated donors compared with Unvaccinated individuals.

Among the tested competitor antigens, PEGylated liposomes most closely resemble the structural characteristics of LNP-based mRNA vaccines. Using PEGylated liposomes as competitors (Figure 3C), Spikevax vaccination resulted in a significantly increased anti-PEG antibody avidity toward all tested liposome formulations compared with Unvaccinated samples. With Comirnaty vaccination, significance was observed only for liposomes containing 2 mol% PEG compared with Unvaccinated samples, while Comirnaty values showed an intermediate trend between Unvaccinated and Spikevax samples across the remaining formulations.

### 3.4. Impact of PEG Chain Length on Anti-PEG IgG Binding

PEG-OH molecules of different chain lengths were used as competitor antigens, and the number of molecules required to achieve 50% inhibition of plasma anti-PEG IgG binding to plate-coated DSPE-mPEG2000 was determined under fixed antibody concentration (2000 ng_eq_/mL). Since hydroxy end-groups are minimally recognized by end-group-specific anti-PEG antibodies, their contribution to PEG binding is likely negligible in this assay [44]. Therefore, the measured inhibition is expected to reflect PEG backbone-specific antibody recognition.

**Figure 4:**
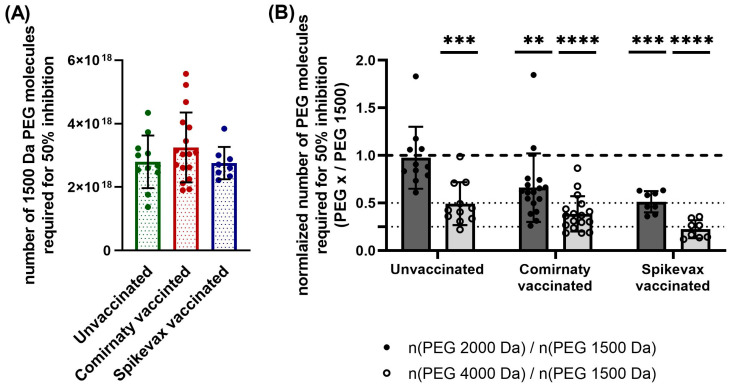
Number of PEG molecules of different lengths as competitor antigens required to achieve 50% inhibition of anti-PEG IgG binding. Numbers (mean ± SD) of 1500 Da PEG molecules required to achieve 50% inhibition were comparable in plasma samples from Unvaccinated (green), Comirnaty-vaccinated (red), and Spikevax-vaccinated (blue) donors (**A**). Numbers (mean ± SD) of PEG molecules required to achieve 50% inhibition of anti-PEG IgG binding, normalized by PEG 1500, for PEG 2000 (dark gray bar with filled circles), and PEG 4000 (light gray bar with open circles) show differences (**B**). Statistical analysis in (**B**) was performed on log-transformed absolute numbers of PEG molecules required to achieve 50% inhibition using two-way repeated-measures ANOVA followed by Šídák’s multiple comparisons test. PEG 1500 Da served as the reference level for post hoc comparisons, which were restricted to PEG 2000 Da and PEG 4000 Da. *p* < 0.05 was considered statistically significant (** *p* < 0.01; *** *p* < 0.001; **** *p* < 0.0001). For multiple-comparison correction, all comparisons across PEG molecular lengths were treated as a single family comprising six pairwise comparisons.

As shown in Figure 4A, similar mean values were observed for 1500 Da PEG-OH across Unvaccinated (2.8 × 10^18^), Comirnaty-vaccinated (3.3 × 10^18^), and Spikevax-vaccinated (2.8 × 10^18^) donors, indicating comparable inhibitory efficiency of PEG-OH of this chain length.

To facilitate comparison across different PEG chain lengths and to better illustrate the relative inhibitory effects of 2000 Da and 4000 Da PEG molecules, the results were normalized to the corresponding 1500 Da PEG value within each sample (Figure 4B). Statistical analyses were performed on log-transformed absolute values using 1500 Da PEG as the reference for all post hoc comparisons.

In samples from Unvaccinated donors, similar numbers of 1500 Da and 2000 Da PEG molecules were required to achieve 50% inhibition (2.8 × 10^18^ vs. 2.7 × 10^18^), whereas significantly lower numbers of 4000 Da PEG molecules (1.3 × 10^18^), approximately half as many, were sufficient to produce the same effect.

In contrast, in plasma samples from Spikevax-vaccinated donors, significantly lower numbers of PEG molecules were required: approximately half as many 2000 Da PEG molecules (1.4 × 10^18^) and about one quarter as many 4000 Da PEG molecules (6.0 × 10^17^) were sufficient to achieve 50% inhibition compared to 1500 Da PEG under the conditions of the competition assay. A similar pattern was observed in Comirnaty-vaccinated donors; however, the reduction was less pronounced than in Spikevax-vaccinated samples.

### 3.5. Antibody Specificity Toward PEG and PS in Unvaccinated and Vaccinated Donors

Repeating oxyethylene (-CH2-CH2-O-) units form the backbone of both PEG and PS molecules; therefore, anti-PEG and anti-PS antibodies exhibit substantial cross-reactivity with these structures [17]. Our results (Figure 3 and Figure 4) demonstrated that anti-PEG IgG antibodies in Unvaccinated and mRNA-LNP-vaccinated donors differ from each other, raising the question of whether vaccination also influences antibody specificity toward PEG versus PS molecules. To investigate this at the population level, ELISA measurements were performed on 325 plasma samples from Unvaccinated and COVID-19-vaccinated donors (shown in Table 1) to quantify antibody binding to PEG and PS antigens using DSPE-mPEG2000- or PS20-immobilized plates, respectively.

**Figure 5:**
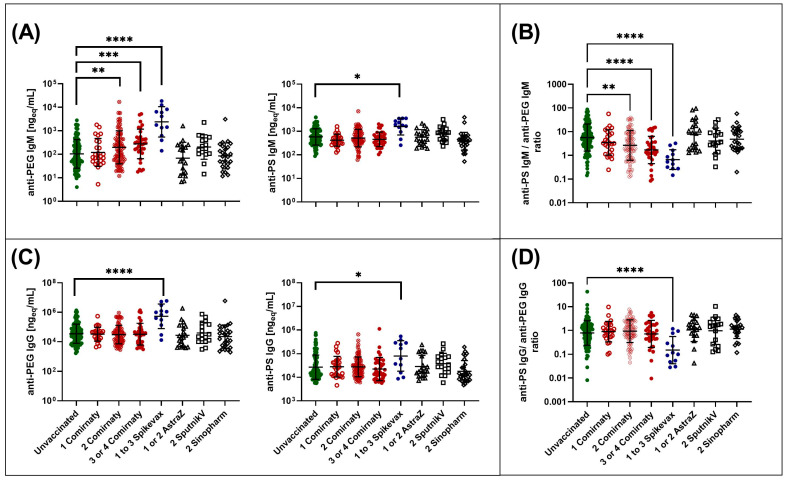
Anti-PEG and anti-PS antibody levels and anti-PS/anti-PEG antibody ratios in Unvaccinated and COVID-19-vaccinated donor samples. Different COVID-19 vaccinations are shown on the *x*-axis, where the numbers (1, 2, 3, and 4) indicate the order of vaccination. Data points represent antibody levels or antibody ratios in samples from Unvaccinated (green), Comirnaty (red), Spikevax (blue), AstraZeneca (empty triangle), Sputnik V (empty square), and Sinopharm (empty diamond) vaccinated donors. Panels (**A**,**C**) show anti-PEG, anti-PS IgM, and IgG antibody levels, respectively. Panels (**B**,**D**) show anti-PS/anti-PEG IgM and IgG ratios, respectively. Log-transformed values are displayed on a logarithmic *y*-axis with back-transformed values shown on the scale. Statistical analysis of panels (**A**,**C**) was performed on log-transformed antibody levels using two-way repeated-measures ANOVA followed by Dunnett’s multiple comparisons test. *p* < 0.05 was considered statistically significant (* *p* < 0.05; ** *p* < 0.01; *** *p* < 0.001; **** *p* < 0.0001). Anti-PEG and anti-PS antibodies were analyzed as separate families, with seven comparisons performed within each family. Statistical analysis of panels (**B**,**D**) was performed on log-transformed antibody ratios using one-way ANOVA followed by Dunnett’s multiple-comparisons test. *p* < 0.05 was considered statistically significant (** *p* < 0.01; **** *p* < 0.0001). For each ratio analysis, all comparisons were treated as a single family comprising seven comparisons.

Figure 5A,C show that anti-PS, anti-PEG IgM, and IgG antibody levels were significantly elevated in plasma samples from individuals vaccinated one to three times with Spikevax, but only anti-PEG IgM levels were significantly higher in donors’ plasma samples vaccinated at least twice with Comirnaty compared with Unvaccinated individuals, which is consistent with previous reports [14].

However, Figure 5B shows that the anti-PS/anti-PEG IgM ratio was significantly lower in most PEGylated LNP-vaccinated groups (individuals vaccinated one to three times with Spikevax, or at least twice with Comirnaty) when compared with Unvaccinated donors. Similarly, Figure 5D demonstrates that the anti-PS/anti-PEG IgG ratio was significantly lower in donors vaccinated with one to three doses of Spikevax compared with Unvaccinated controls. Figure 5A,C show that the decreased anti-PS/anti-PEG ratio is primarily associated with a greater increase in anti-PEG antibody levels than in anti-PS antibody levels following PEGylated LNP vaccination.

In addition, although some tendencies toward increased PS specificity could be observed, no significant shifts in anti-PS/anti-PEG IgM or IgG ratios were observed in recipients of PS80-containing vaccines (AstraZeneca or Sputnik V) or in recipients of vaccines containing neither PEG nor PS (Sinopharm), compared with Unvaccinated donors.

## 4. Discussion

Although polyethylene glycol (PEG) is widely used in nanomedicine and drug delivery systems, its immunological safety has become an increasing concern in recent years. Anti-PEG antibodies are recognized contributors to hypersensitivity reactions associated with PEGylated therapeutics and PEG-containing pharmaceutics [16,32,33,34,35,36,41,42,48,49,50,51,52,53]. Clinical studies have reported a higher incidence of immediate hypersensitivity reactions following PEGylated vaccination, particularly after booster doses and more frequently after Spikevax compared with Comirnaty [32,33,34,35,36,37,54]. In addition, an experimental study reported immediate anaphylaxis in anti-PEG hyper-immune pigs following administration of Comirnaty [55].

PEGylated mRNA-LNP-based COVID-19 vaccines have been shown to induce elevated levels of anti-PEG antibodies in humans [14,22,23,24,25]. The shared poly(oxyethylene) moieties of PEGs and PSs lead to substantial cross-reactivity between anti-PEG and anti-PS antibodies [15,16,17]. Despite the expanding clinical use of PEGylated and PS-containing medicines, the fundamental properties of anti-poly(oxyethylene) antibodies remain incompletely characterized. In particular, limited information is available regarding how the affinity and specificity of these antibodies may change following widespread exposure to PEGylated mRNA vaccines. The present study aims to address this gap.

### 4.1. Binding Properties of Naturally Occurring Anti-PEG Antibodies

Most studies focus on treatment-induced anti-PEG antibodies generated following exposure to PEG-containing agents. In contrast, antibodies detected in Unvaccinated donors without documented exposure to PEG-containing therapeutics represent pre-existing or naturally occurring anti-PEG antibodies, likely arising from environmental exposure to PEG- or PS-containing consumer products via percutaneous or intestinal routes [56].

To investigate whether naturally occurring anti-PEG IgG antibodies exhibit end-group preferences, we compared their avidity toward DSPE-mPEG2000 and PS20 micelles representing methoxy- and hydroxy-terminated PEG structures, respectively. No significant difference was observed between DSPE-mPEG2000 and PS20 micelles (Figure 1), despite DSPE-mPEG2000 micelles being larger [46,47] and having methoxy end-groups, which are preferentially recognized by anti-PEG antibodies over the hydroxy end-group of PS20 [44,45]. This suggests that naturally occurring anti-PEG IgG antibodies have a limited capacity to discriminate between different PEG end-group chemistries and predominantly recognize the PEG backbone. These findings are further supported by the analysis of *K/q* values (Figure 2), which showed no significant difference in relative anti-PEG IgG avidity between free hydroxy-terminated PEG molecules and methoxy-terminated PEG chains presented on a micelle. However, it must be noted that DSPE-mPEG2000 and PS20 micelles also differ in hydrodynamic size (approximately 16.8 nm vs. 7.2 nm, respectively), indicating that equivalent *K* values may not be attributed to end-group recognition alone.

It was also demonstrated that the avidity of these naturally occurring anti-PEG antibodies increased with the size of PEG-containing antigens (Figure 1), reflecting the presence of multiple accessible epitopes that enable multivalent antibody engagement and thereby enhance functional avidity rather than intrinsic affinity [11,57]. Additionally, PEG chains anchored to the liposomal surface via their hydrophobic lipid moieties adopt a more ordered outward orientation compared with freely diffusing PEG molecules. Such spatial confinement restricts polymer conformational freedom and may reduce the entropic penalty associated with antibody binding, thereby facilitating antigen–antibody interactions, as described for protein adsorption on surfaces with grafted polymers [58].

In PEGylated liposomes, increased epitope density did not proportionally enhance the avidity of antibodies (*K*; Figure 1): although 10 mol% PEG liposomes contained substantially more PEG molecules than 2 mol% liposomes, overall avidity remained comparable. Moreover, the normalized avidity per PEG molecule (*K/q*; Figure 2) was in reverse ratio with the surface PEG density of liposomes, which likely reflects differences in PEG chain conformation on the liposome surface. Based on previous studies of PEGylated liposomes with similar PEG surface densities, PEG chains at 2 mol% PEG are expected to predominantly adopt a mushroom conformation, whereas increasing PEG density has been reported to promote a mushroom–brush transition (5 mol%) and eventually a dense brush regime at 10 mol% PEG [59,60]. If such conformational changes occur in the present liposome formulations, in the dense brush regime, steric crowding, constrained PEG chain orientation, and restricted chain flexibility could reduce antibody accessibility to PEG backbones [61]. In contrast, the more accessible and flexible PEG chains in the mushroom regime facilitate more effective antibody engagement at the level of individual PEG molecules (Figure 2). The reduced per-chain interaction is compensated for by the higher number of PEG epitopes on highly PEGylated liposomes.

### 4.2. Vaccination-Induced Remodeling of Anti-PEG Antibody Recognition

In agreement with previous studies [14,22,23,24,25], PEGylated mRNA-LNP vaccination significantly increased anti-PEG IgM and IgG levels, along with anti-PS antibody levels (Figure 5). However, PEG- and PS-specific antibody levels did not increase proportionally; instead, mRNA-LNP vaccination altered the relative binding preference of the antibody population toward DSPE-mPEG2000 compared with PS20 (Figure 5). This indicates that poly(oxyethylene) induced antibodies are not uniform, but exhibit differential binding to DSPE-mPEG2000- and PS20-coated antigens. Accordingly, Spikevax and Comirnaty vaccination appeared to shift antibody binding preferences toward DSPE-mPEG2000 relative to PS20. This observation is consistent with the structural similarity between the PEG-lipids present in the vaccine formulations and DSPE-mPEG2000; however, the present assay does not allow identification of the specific molecular features responsible for this shift in binding preference.

This interpretation is further supported by previous studies demonstrating that methoxy-PEG-containing immunogens can induce antibodies with preferential binding to methoxy-terminated PEG compared to hydroxy-terminated analogs [44,45]. In contrast, samples from individuals vaccinated with PS80-containing vaccines (Sputnik V, AstraZeneca) did not show significant differences in anti-PEG antibody levels [14] or in the PS/PEG-specific antibody ratio (Figure 5), suggesting that PS80 is substantially less immunogenic than mPEG2000. It should also be noted that while PEG chains are covalently attached to the anchor lipid in LNP vaccines, PS80 is present as a soluble additive. This difference in presentation likely contributes to the observed differences in immunogenicity and further supports that antigen anchoring and mode of presentation, rather than the mere presence of poly(oxyethylene) units, are key determinants of the immune response.

As discussed above, pre-existing anti-PEG IgG exhibited minimal ability to discriminate between PEG end-groups. In contrast, antibodies in PEGylated LNP-vaccinated samples showed higher avidity toward methoxy-terminated DSPE-mPEG2000 micelles without significantly affecting avidity toward hydroxy-terminated PS20 micelles (Figure 3B) in accordance with previous reports [44,45]. Increased avidity of anti-PEG IgG antibodies from vaccinated donors was also observed toward liposomes (Figure 3C), including those with a dense-brush PEG conformation (10 mol% PEG), which, among the tested antigens, most closely resembles PEGylated LNP vaccines, where PEG end-groups are most accessible for antibody binding [8,62]. The enhanced recognition of methoxy end-groups indicates the induction of methoxy end-group-specific anti-PEG antibodies. Since PEG backbone-specific and end-group-specific antibodies originate from different B-cell populations, this finding suggests that PEGylated vaccines may promote the induction of B-cell clones targeting methoxy-terminated PEG epitopes.

To further elucidate structural determinants of antibody recognition, we analyzed the PEG backbone length required to accommodate antibody binding using hydroxy-terminated PEG molecules as competitor antigens. A reduction in the number of competitor molecules required for 50% inhibition may reflect at least two, not mutually exclusive, mechanisms: stronger binding of individual PEG molecules to anti-PEG antibodies, or the ability of longer PEG chains to engage multiple antibody paratopes simultaneously. Structural studies have shown that a single Fab paratope typically binds approximately 700 Da (15–16 oxyethylene units) of PEG [63,64]; however, considering steric constraints and the spatial requirements of antibody binding, simultaneous engagement of two paratopes by a single 1500 Da PEG chain was unlikely. This implies that, in our measurements, a 1500 Da PEG chain can bind only one anti-PEG IgG paratope, independent of whether the antibodies are pre-existing or vaccine-induced (Figure 4A). Moreover, in the case of Unvaccinated donors, similar amounts of 1500 Da and 2000 Da PEG-OH molecules were required to achieve 50% inhibition of antibody binding (Figure 4B), suggesting comparable binding behavior between the two PEG chain lengths under the tested conditions. In contrast, analysis of samples from Spikevax-vaccinated donors showed that approximately half the amount of 2000 Da PEG-OH was sufficient to achieve 50% inhibition relative to 1500 Da PEG-OH. One possible interpretation is that 2000 Da PEG-OH can accommodate binding of two vaccine-induced antibody paratopes, either from a single IgG molecule or from two separate IgG molecules, thereby increasing its competitive efficiency. Analyzing the inhibitory properties of 4000 Da PEG-OH (containing 91 oxyethylene units), a similar interpretation can be proposed, namely that while this PEG length may accommodate approximately two naturally existing anti-PEG IgG antibody paratopes on average, it may engage a greater number (up to four or five) of Spikevax-induced anti-PEG antibody paratopes under the condition of the competition assay (Figure 4B).

These findings indicate that, following vaccination, shorter poly(oxyethylene) segments are sufficient for effective anti-PEG IgG binding, or alternatively, that vaccine-induced antibodies exhibit altered PEG chain-length dependence in this competition assay, consistent with a narrowing of the minimal epitope required for antibody recognition. This shift toward recognition of shorter PEG segments may further enhance antibody binding to densely PEGylated nanoparticle surfaces, where only limited portions of individual PEG chains are sterically accessible. However, the present data do not allow discrimination between increased per-molecule binding efficiency and multivalent paratope engagement as the primary mechanism underlying the observed reduction in competitor requirement. Therefore, the proposed structural interpretation should be regarded as a working hypothesis, and further structural or kinetic studies would be required to establish the underlying mechanism.

### 4.3. Immunological and Clinical Implications of Affinity-Matured Anti-PEG Antibodies

Our results demonstrate differences in the binding characteristics of anti-PEG antibodies in samples from individuals receiving PEGylated lipid nanoparticle (LNP)-based formulations compared with Unvaccinated individuals, including increased recognition of methoxy end-groups and shorter PEG backbone segments, consistent with a binding pattern compatible with multivalent engagement along a single PEG chain. In addition, in samples following PEGylated vaccination, antibody avidity toward almost all tested PEG structures was increased (Figure 3), which cannot be explained solely by the recognition of shorter PEG chain segments. Consequently, affinity maturation of pre-existing PEG-reactive clones and the expansion of higher-avidity vaccine-associated antibody populations are likely to contribute. In parallel, the emergence of increased methoxy end-group specificity suggests the induction of new B-cell clones with preferential recognition of methoxy-terminated PEG epitopes.

PEG antigens consist of highly repetitive structural units and can efficiently cross-link B-cell receptors, typically triggering T-cell-independent type II immune responses, in which classical affinity maturation has traditionally been considered limited [65]. Our data nevertheless suggest that affinity maturation of anti-PEG antibodies or alternative mechanisms such as clonal selection or expansion of pre-existing high-avidity clones may occur following PEGylated LNP vaccination. Elucidating the mechanisms underlying this process will be critical for understanding, predicting, and ultimately preventing the harmful effects associated with anti-PEG antibodies.

As demonstrated by our results (Figure 3, Figure 4 and Figure 5), differences in antibody binding preferences were more pronounced in samples from Spikevax-vaccinated individuals than in samples from Comirnaty-vaccinated individuals, whereas greater inter-individual variability was observed in the Comirnaty-vaccinated group. This difference is likely attributable to the higher PEG-lipid dose administered with Spikevax (approximately 117 µg per dose) compared with Comirnaty (approximately 50 µg per dose) [8], which may provide a stronger immunogenic stimulus. However, given the limited size of the Spikevax cohort, we cannot determine whether this reflects a true biological difference in variability or sampling effects.

The emergence of high-avidity anti-PEG IgG antibodies with enhanced backbone and end-group recognition, capable of multivalent engagement of densely PEGylated nanoparticle surfaces, may have potential clinical relevance. Previous studies have suggested that anti-PEG antibodies can contribute to infusion-related hypersensitivity reactions and anaphylaxis observed in a subset of vaccinated individuals [14,20,23]. While the present study did not assess clinical outcomes or immunological effector mechanisms, the observed increase in antibody avidity may be consistent with enhanced immune recognition of PEGylated nanomedicines. Based on mechanisms proposed in the literature, higher-avidity anti-PEG antibodies could theoretically promote more efficient complement activation and enhanced opsonization of lipid nanoparticles [26,27,28]. In addition, the stronger complement activation may be associated with enhanced opsonization of lipid nanoparticles. This, in turn, may promote their recognition by phagocytic cells and contribute to accelerated blood clearance (ABC), thereby altering vaccine pharmacokinetics and possibly immunogenicity. However, these possibilities were not directly investigated in the present study and require further experimental and clinical validation.

Taken together, our study suggests that the immunological response to PEG-containing therapeutics may differ in populations previously exposed to PEGylated mRNA-LNP vaccines compared with patterns reported before their widespread use. To our best knowledge, for the first time in the literature, our findings point to an unfulfilled need for avidity- and specificity-aware immunogenicity assessment of PEGylated therapeutics and argue for careful consideration of PEG use in repeated nanomedicine treatments, particularly in populations with prior exposure to PEG-LNP formulations. A deeper understanding of the mechanisms governing anti-PEG antibody affinity maturation may ultimately improve risk prediction and guide the development of safer PEG-containing therapeutics.

### 4.4. Limitations of the Study

Several limitations of this study should be acknowledged. First, the avidity cohort was relatively small and unbalanced across vaccine groups, particularly for Spikevax recipients, and the assay requirements restricted the analysis to samples containing sufficiently high anti-PEG IgG levels, which may limit the representativeness of the results for individuals with very low antibody concentrations. Second, blood samples were collected at different time points after vaccination, and a limited number of donors contributed more than one sample during the study period. Because these repeated samples were obtained at irregular intervals and did not constitute a balanced longitudinal dataset, analyses were performed according to vaccination-status groups rather than using a repeated-measures design. Consequently, the cross-sectional nature of the study does not allow definitive causal attribution of the observed antibody characteristics to vaccination alone. Third, the grouping strategy was influenced by the available sample numbers, resulting in asymmetric vaccine subgroups and the absence of certain dose-specific categories. Finally, the present study focused primarily on anti-PEG IgG antibodies. The presence of other antibody isotypes in plasma, the choice of coating antigen, the use of model PEGylated liposomes that differ from the composition of mRNA-LNP vaccines, and the absence of a vehicle-matched control group may also influence the measured avidity values and their biological interpretation.

## Figures and Tables

**Table 1:** Definition of donor groups used for statistical analysis of antibody specificity against PEG and PS. Donor groups used in statistical analyses are shown in Column 1. Each group represents a merged category of vaccination regimens detailed in Column 2. Column 3 indicates the number of donor samples in each subgroup. For each donor and vaccination status, only one sample was included; repeated measurements from the same donor at the same vaccination status were not counted multiple times.

Groups for Statistical Analysis	Number and Type of Vaccinations	Number of Donors’ Samples
Unvaccinated	-	114
1 Comirnaty	1 Comirnaty	27
2 Comirnaty	2 Comirnaty	72
3 or 4 Comirnaty	3 Comirnaty	32
	4 Comirnaty	3
1 to 3 Spikevax	1 Spikevax	2
	2 Spikevax	7
	3 Spikevax	3
1 or 2 AstraZeneca	1 AstraZeneca	6
	2 AstraZeneca	14
2 Sputnik V	2 Sputnik V	17
2 Sinopharm	2 Sinopharm	28

**Table 2:** Composition of liposomes used as competitor antigens. Molar ratio of the liposome-forming components, final concentration of lipids, and average size [nm] of the different liposomes are shown. Lipid compositions labeled as 2, 5, and 10 mol% PEG refer to nominal target formulations.

Competitor Molecules		Molar Ratio [%]			Final Lipid Concentration [mg/mL]	Mean Size (Diameter) [nm]
		Chol	DSPE-mPEG2000	HSPC		
Conventional liposome		38.24	-	61.75	13.63	102.4 ± 0.5
PEGylated liposomes	2 mol% PEG	38.38	2.30	59.32	14.58	94.7 ± 0.6
	5 mol% PEG	38.18	5.26	56.56	15.96	82.5 ± 0.6
	10 mol% PEG	37.69	11.37	50.94	18.91	69.3 ± 0.5

**Table 3:** Structural characteristics of competitor antigens, including the number of oxyethylene units, PEG content, and final concentration ranges used in ELISA equilibrium titration experiments. PEG-related molecules per structure (*q*) were calculated based on the actual DSPE-mPEG2000 molar ratio content of the liposomes.

Competitor Antigen		Structure	Oxyethylene Units per PEG Molecule	Estimated PEG-Related Molecule per Structure (*q*)	Concentration [nM]
PEG1500		Single molecule with –OH end-group	34	1	1.28–100,000
PEG2000			45	1	1.28–100,000
PEG4000			91	1	1.28–100,000
PS20		Micelle	5/20 ^b^	70	8.19–64,000 ^a^
DSPE-mPEG2000			45	60	4.55–2777 ^a^
Conventional liposome		Liposome	-	-	0.0006–11.6 ^a^
PEGylated liposome	2 mol% PEG		45	2282	0.0062–13.6 ^a^
	5 mol% PEG		45	3922	0.0083–18.1 ^a^
	10 mol% PEG		45	5895	0.0121–26.4 ^a^

^a^ The concentrations (nM) represent the calculated concentrations of micelles or liposomes. ^b^ The numbers are the average linear number/total number of oxyethylene units per PS20 molecules.

## Data Availability

The data presented in this study are available from the corresponding author upon reasonable request. The data are not publicly available due to privacy and ethical restrictions related to human participant data.

## Supplementary Materials

The following supporting information can be downloaded at: https://www.mdpi.com/article/10.3390/pharmaceutics18070815/s1, Table S1: Demographic and immunological characteristics of the donor cohort included in the anti-PEG antibody avidity measurements. Figure S1: Effect of conventional liposomes in anti-PEG antibody competition measurements.
