# Supplementary material for: Anti-PEG Immunogenicity of mRNA-LNP Vaccines in Humans: Evidence for Population-Level Changes in the Anti-PEG Antibody Repertoire
Source: Pharmaceutics. 2026 Jun 30;18(7):815. doi: 10.3390/pharmaceutics18070815 (PMC13414645; doi:10.3390/pharmaceutics18070815)
Supplement: Supplementary file 1 [file pharmaceutics-18-00815-s001.zip › pharmaceutics-4334499-supplementary.pdf]

Supplementary Table S1. **Demographic and immunological characteristics of the donor cohort included in the anti-PEG antibody avidity measurements.** The table summarizes the characteristics of the samples selected for ELISA equilibrium titration analysis of anti-PEG antibody avidity, including vaccination status, anti-PEG IgM and IgG concentrations, donor sex, donor age, and the time between the last COVID-19 vaccination and blood withdrawal. Samples were selected from the larger study cohort based on the availability of sufficient plasma volume and anti-PEG IgG concentrations required for reliable competitive ELISA measurements.

**Sample**	**Plazma anti-PEG antibody level [ng/ml]**		**Donor's Vaccination status**	**Days between last vaccination and blood withdrawal**	**Donor's Sex**	**Donor's Age**
	IgM	IgG				
1	1363	620 411	2 Spikevax	24	M	51
2	8201	2 484 810	2 Spikevax	155	F	55
3	1777	1 137 285	2 Spikevax	78	F	59
4	5018	961 789	2 Spikevax	178	M	40
5	18635	6 225 245	2 Spikevax	523	F	24
6	3248	3 038 602	3 Spikevax	127	M	30
7	3635	186 969	3 Spikevax	231	M	28
8	5866	772 614	1 Spikevax	30	F	30
9	2565	460 105	-	-	F	46
10	153	251 841	-	-	F	21
11	47	1 050 943	-	-	F	54
12	36	128 129	-	-	F	31
13	52	200 984	-	-	M	31
14	50	138 354	-	-	M	8
15	399	1 067 858	-	-	F	41
16	34	21 937	-	-	F	30
17	747	79 284	-	-	F	10
18	109	76 091	-	-	F	42
19	591	192 001	-	-	F	33
20	37	461 470	1 Comirnaty	16	F	49
21	279	406 140	2 Comirnaty	81	F	26
22	761	235 814	1 Comirnaty	21	M	16
23	705	455 226	2 Comirnaty	27	F	30
24	131	506 558	2 Comirnaty	152	M	46
25	62	873 759	3 Comirnaty	23	F	59
26	327	396 602	3 Comirnaty	26	F	27
27	1104	725 800	2 Comirnaty	251	F	24
28	160	345 140	2 Comirnaty	167	F	38
29	171	259 916	3 Comirnaty	28	M	77
30	17049	220 962	2 Comirnaty	125	M	24
31	428	332 752	2 Comirnaty	165	F	44
32	624	673 616	3 Comirnaty	40	F	24
33	644	83 320	1 Comirnaty	33	F	10
34	1393	161 235	4 Comirnaty	16	F	63
35	651	200 403	3 Comirnaty	654	F	33
36	41	988 196	1 Comirnaty	30	F	23

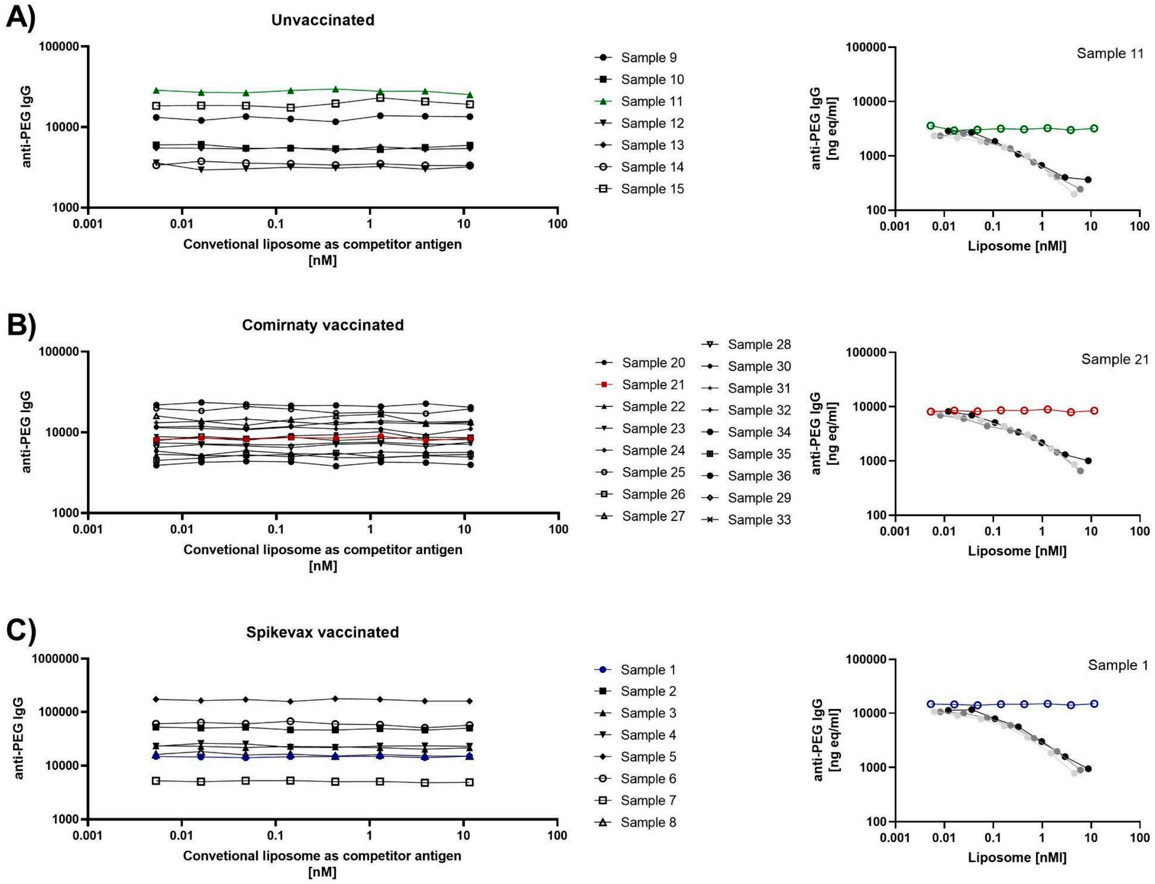

Supplementary Figure S1. Effect of conventional liposomes in anti-PEG antibody competition measurements. Anti-PEG antibody concentrations were measured in the presence of conventional liposomes lacking PEG across all samples. Data are shown separately for Unvaccinated (A), Comirnaty-vaccinated (B), and Spikevax-vaccinated (C) donor groups. No effect on anti-PEG IgG levels was observed, confirming that conventional liposomes do not interfere with antibody detection. In addition, representative sample data from each study group are shown to illustrate the comparison between conventional liposomes and PEG-containing liposomes (2, 5, and 10 mol% PEG) used as competitor antigens. While conventional liposomes showed no competitive effect, PEG-containing liposomes induced a concentration-dependent decrease in measured anti-PEG IgG levels, demonstrating assay specificity.
